# Amblyomin-X induces ER stress, mitochondrial dysfunction, and caspase activation in human melanoma and pancreatic tumor cell

**DOI:** 10.1007/s11010-016-2683-4

**Published:** 2016-03-25

**Authors:** Katia L. P. Morais, Mario Thiego Fernandes Pacheco, Carolina Maria Berra, Rosemary V. Bosch, Juliana Mozer Sciani, Roger Chammas, Renata de Freitas Saito, Asif Iqbal, Ana Marisa Chudzinski-Tavassi

**Affiliations:** Biochemistry and Biophysics Laboratory, Butantan Institute, Av. Vital Brazil, 1500, São Paulo, SP 05503-900 Brazil; Department of Biochemistry, Federal University of São Paulo, São Paulo, SP Brazil; Biochemistry Department, Institute of Chemistry, University of São Paulo, São Paulo, Brazil; Experimental Oncology Medical Investigation Laboratory – LIM/24, University of São Paulo School of Medicine, São Paulo, SP Brazil

**Keywords:** Amblyomin-X, Kunitz-type inhibitor, Proteasome inhibitor, Endoplasmic reticulum stress, Antitumor drug candidate

## Abstract

During the last two decades, new insights into proteasome function and its role in several human diseases made it a potential therapeutic target. In this context, Amblyomin-X is a Kunitz-type FXa inhibitor similar to endogenous tissue factor pathway inhibitor (TFPI) and is a novel proteasome inhibitor. Herein, we have demonstrated Amblyomin-X cytotoxicity to different tumor cells lines such as pancreatic (Panc1, AsPC1BxPC3) and melanoma (SK-MEL-5 and SK-MEL-28). Of note, Amblyomin-X was not cytotoxic to normal human fibroblast cells. In addition, Amblyomin-X promoted accumulation of ER stress markers (GRP78 and GADD153) in sensitive (SK-MEL-28) and bortezomib-resistant (Mia-PaCa-2) tumor cells. The intracellular calcium concentration [Ca^2+^]_*i*_ was slightly modulated in human tumor cells (SK-MEL-28 and Mia-PaCa-2) after 24 h of Amblyomin-X treatment. Furthermore, Amblyomin-X induced mitochondrial dysfunction, cytochrome-c release, PARP cleavage, and activation of caspase cascade in both human tumor (SK-MEL-28 and Mia-PaCa-2) cells. These investigations might help in further understanding of the antitumor properties of Amblyomin-X.

## Introduction

 The proteasome is a large protein complex responsible for regulation of intracellular proteolysis in all eukaryotic cells. Thereby, it plays an essential role in the regulation of the cellular processes, including protein quality control, cell cycle progression, differentiation, signal transduction, apoptosis, gene expression, and antigen processing [1–3]. Thus, a dysfunction of proteasome activity has detrimental effect on cell homeostasis. In humans, proteasome deregulation has numerous implications in pathologies such as cancer, autoimmune diseases, neurodegenerative diseases, and viral infections [1–3], rendering it as a potential therapeutic target [2]. A proteasome inhibitor bortezomib is approved by FDA for the treatment of multiple myeloma, and several others are in clinical trials [3].

Cell death induced by proteasome inhibitors could involve endoplasmic reticulum (ER) stress [4–7]. Important steps in the synthesis of the proteins directed to the secretory pathway or to plasma membrane are processed in the ER-lumen, such as folding and post-translational modifications [8, 9]. However, if correct folding is not reached, the first quality control system (calnexin/calreticulin) works to bring proteins in the correct folding [8]. Many misfolded proteins undergo this quality control system and can be eliminated via the proteasome, by a mechanism known as endoplasmic reticulum-associated protein degradation (ERAD) [10, 11]. The inhibition of this process ultimately leads to the accumulation and aggregation of proteins in the ER and the induction of ER stress [8, 11]. Thus, ERAD fine-tuning is essential for proteostasis [10]. Despite no direct association, ERAD could share common pathways with unfolded protein response (UPR), for instance, IRE1-dependent transcription factor XBP1 codes for several ERAD-related genes [10, 12]. Overall, those pathways underline key ubiquitin–proteasome system functions related to un/misfolded protein disposal and cell stress response.

During UPR activation, the chaperone 78 kDa glucose-regulated protein (GRP78) is released from three ER transmembrane proteins: protein kinase-like ER kinase (PERK); the inositol requiring kinase 1 alpha (IRE1α); and the transcription factor-activating transcription factor 6 (ATF6) [13, 14]. The ER transmembrane proteins IRE1α and PERK undergo dimerization and auto-phosphorylation and are consequently activated that redirect alternative transcription of chaperones and transcription factors, such GRP78 and factor-activating transcription factor 4 (ATF4) [15]. PERK phosphorylates the eukaryotic translation initiation factor (eIF)2α and results in a global attenuation of protein translation. These events help the cell to cope with accumulated unfolded proteins and restore ER homeostasis. In parallel, ATF6 is transported to the Golgi, where it is sequentially cleaved by Golgi resident proteases, resulting in the release of cytosolic domain of ATF6, which goes to the nucleus to activate the transcription of target genes (GRP78, GADD153, calreticulin) [16]. However, failure to relieve prolonged or severe ER stress, triggers apoptosis [8].

If ER stress is persistent or severe and ER homeostasis is not restored, UPR activates cell death pathways. One of ER stress-induced cell death pathways is mediated by IRE1 that can interact with the adaptor protein of TNF receptor-associated factor 2 (TRAF2), leading to the activation of apoptosis signal-regulating kinase 1 [17]. This might mediate apoptosis in response to ER stress by activation of c-Jun-*N*-terminal kinase (JNK). The complex IRE1α/TRAF2 also activates ER-resident caspase-4 [17]. Furthermore, ATF4 can lead to the upregulation of nuclear transcription factors of growth arrest and DNA damage (GADD153). This able cells to (i) transcriptionally down-regulate the levels of Bcl-2 and upregulate DR5; (ii) cause cell cycle arrest; (iii) sensitize the mitochondria to pro-apoptotic Bcl-2 family proteins; (iv) lead to a depletion of the cellular glutathione levels and increase levels of reactive oxygen species [18–20]. In addition, calcium released from the ER is rapidly taken up by the mitochondria, where it leads to collapse of inner membrane potential, cytochrome-c release, caspase cascade activation, and subsequent initiation of apoptosis [21–23].

In this context, Amblyomin-X is a homolog of Kunitz-type protein identified in the transcriptome of the salivary glands from the adult *Amblyomma cajennense* tick [24, 25]. The recombinant protein form of Amblyomin-X has presented antitumor activity via induction of apoptosis and inhibition of proteasome [26, 27]. The human melanoma (SK-MEL-28) and human pancreas adenocarcinoma (Mia-PaCa-2) tumor cells were a good choice to investigate the mechanism of action of Amblyomin-X, because both of them are sensitive to pro-apoptotic effects of Amblyomin-X [24]. In addition, Mia-PaCa-2 cells are resistant to bortezomib-induced apoptosis [28]. In this study, we reported pro-apoptotic effect of Amblyomin-X in these human tumor cells associated to inhibition of proteasome function, ER stress (UPR markers upregulation), mobilization of [Ca^2+^]_*i*_, mitochondrial dysfunction, PARP cleavage, and caspase-3 activation. Interestingly, none of these changes were observed in normal human fibroblast cells.

## Results

### Amblyomin-X induces cytotoxicity in human tumor cells

The effect of Amblyomin-X (1 µM) on cell viability of human melanoma (SK-MEL-28 and SK-MEL-5) and pancreatic (Mia-PaCa-2, Panc1, AsPC1, and BxPC3) tumor cells was investigated. After 48 h, cells treated with Amblyomin-X decreased the viability considerably as shown in Table 1. The amount of viable cells was different for both tumors cells; for example, ~67 % of Mia-PaCa-2 and ~29 % of metastatic-derived AsPC1 were viable after 48 h of Amblyomin-X treatment (Table 1). However, no changes in cell viability of human fibroblast were observed after 48 h of Amblyomin-X treatment (Table 1).

**Table 1:** Effect of Amblyomin-X in cell viability of different tumor cell lines

Tumor cell line	Origin	Viability
Mia-PaCa-2	Human pancreatic adenocarcinoma	67.02 ± 5.9***
Panc1	Human pancreatic epithelioid carcinoma	35.81 ± 1.2**
AsPC1	Human pancreatic adenocarcinoma	29.56 ± 1.03**
BxPC3	Human pancreatic adenocarcinoma	13.89 ± 0.50**
SK-MEL-28	Human melanoma	43.91 ± 10.5**
SK-MEL-5	Human melanoma	42.34 ± 13.75**
Human Fibroblast	Non-tumor cell	106.76 ± 10.01

Tumor cells lines were incubated with 1 µM of Amblyomin-X for 48 h, and cell viability was assessed as described in Materials and Methods. The data presented as the mean ± SD of three independent experiments

**p ≤ 0.005, ***p ≤ 0.001 compared with the control group (corresponding untreated cells), as determined by one-way ANOVA

### Amblyomin-X triggers ER stress in tumor cells

It has been reported that Amblyomin-X inhibits proteasome system [26, 27]. The inhibition of proteasome can cause ER stress, thus, we decided to assess whether Amblyomin-X could induce ER stress. A representative of human melanoma and pancreatic tumor cells lines i.e., Mia-PaCa-2 and SK-MEL-28 were selected for this study, since the effect of Amblyomin-X on the protein clearance in those tumor cells has been investigated [29]. Human fibroblast were used as non-tumor cell model.

**Fig. 1:**
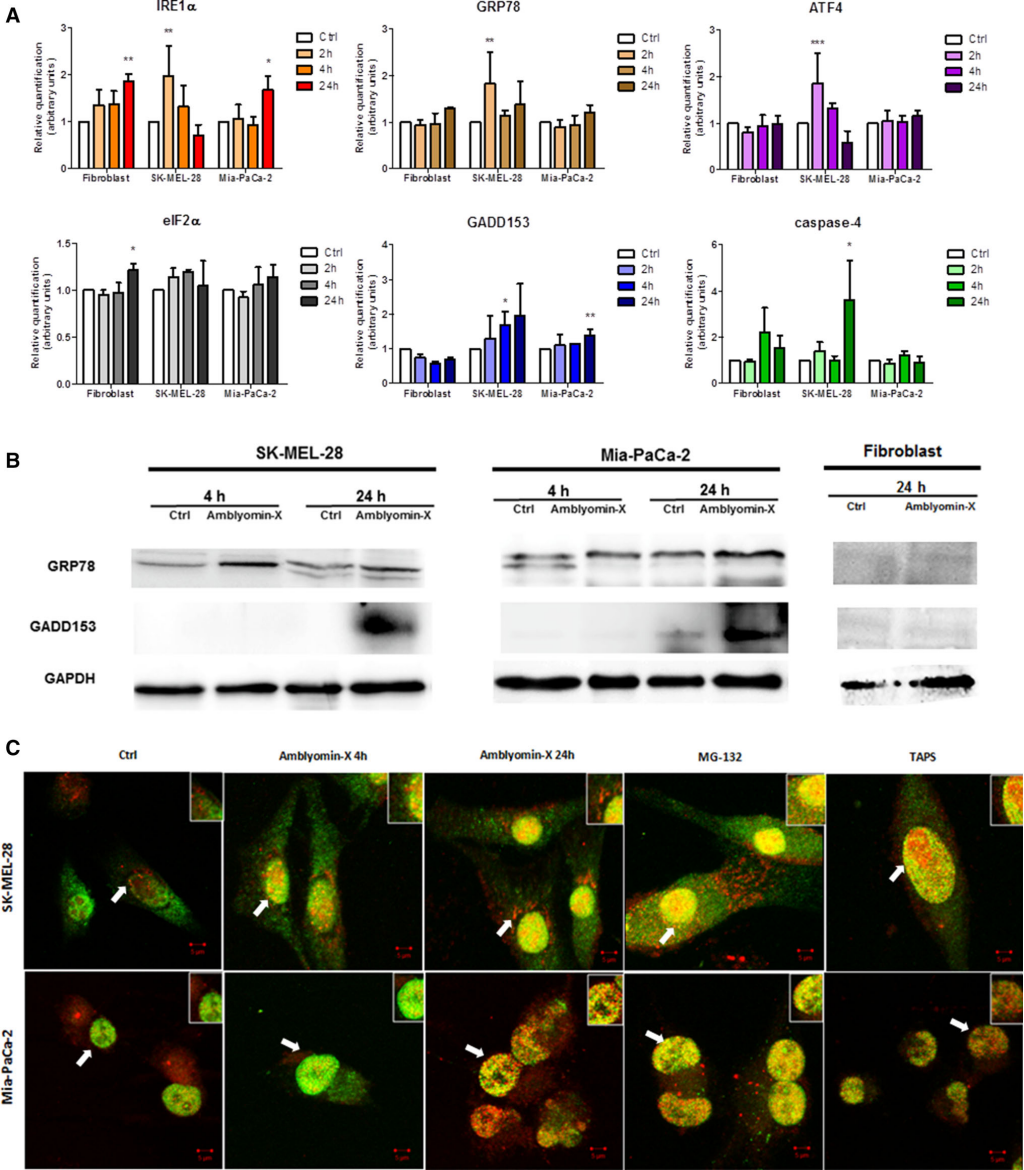
Amblyomin-X induces ER stress in tumor cells. **a** Cells were treated with Amblyomin-X (0.5 µM) at the indicated times and levels of gene expression of targets related to ER stress were evaluated by real-time PCR. **b** After the indicated period of treatment, cells were lysed with RIPA buffer and 30 µg of total protein was used for the tests. Western blot analysis was performed by checking anti-GRP78, anti-GADD153, and anti-GAPDH (endogenous controls). **c** Confocal microscopy analysis of indirect immunofluorescence of ATF-6α and ERGIC53. Cells treated with vehicle (PBS), or with 0.5 µM of Amblyomin-X for 4 or 24 h, or with MG-132 (2.5 µM) and TAPS (1 µM) for 24 h each. The final overlay image is a representative of three independent experiments in which *red fluorescence* represents ATF-6α, while *green fluorescence* represents ERGIC53, and *yellow* corresponds to merging of both *red and green*. Values are mean ± SD of three independent experiments. *p ≤ 0.05; **p ≤ 0.01, and ***p ≤ 0001. (Color figure online)

The mRNA levels of the proteins related to the ER stress were estimated using real-time PCR. When SK-MEL-28 cells are treated with 0.5 µM of Amblyomin-X, the mRNA levels of IRE1α, ATF4, and GRP78 genes increased after 2 h and only GADD153 gene expression augmented in a time-dependent manner but was significant only after 4 h (Fig. 1a). The expression of the caspase-4 gene increased after 24 h of treatment with Amblyomin-X. On the other hand, the expression of the eIF2α gene did not increase in the SK-MEL-28 cells treated with 0.5 µM Amblyomin-X (Fig. 1a). In Mia-PaCa-2 cells treated with 0.5 µM of Amblyomin, the expression of IRE1α and GADD153 genes increased after 24 h (Fig. 1a). The GRP78, ATF4, eIF2α, and caspase-4 genes showed no significant changes in expression when compared to their corresponding controls. Moreover, we verified the protein levels of GRP78 and GADD153 in tumor cells after Amblyomin-X treatment. The GRP78 protein levels increased in SK-MEL-28 after 4 h and in Mia-PaCa-2 after 24 h (Fig. 1b). The GADD153 protein levels increased after 24 h in both cells lines (Fig. 1b). Human fibroblast cells showed some alterations in IRE1α and eIF2α gene after 24 h of Amblyomin-X treatment (Fig. 1b). In contrast, no alterations were found in GRP78 and GADD153 protein levels in human fibroblast after Amblyomin-X treatment.

It is well established that ATF-6α and cargo proteins are transported from the ER to the Golgi apparatus through the ER-Golgi intermediate compartment (ERGIC) [30, 31] and ERGIC-53 is a specific marker for ERGIC [31]. As shown in Fig. 1c, anti-ATF-6α antibody stained cytoplasmic in “Ctrl” tumor cells. In contrast, ATF-6α moved toward and reached the nucleus of tumor cells treated with Amblyomin-X. Importantly, ATF6 staining overlapped with ERGIC-53, and the images suggest close proximity to nucleus.

### Amblyomin-X modulates mobilization of [Ca^2+^]_i_ in tumor cells

**Fig. 2:**
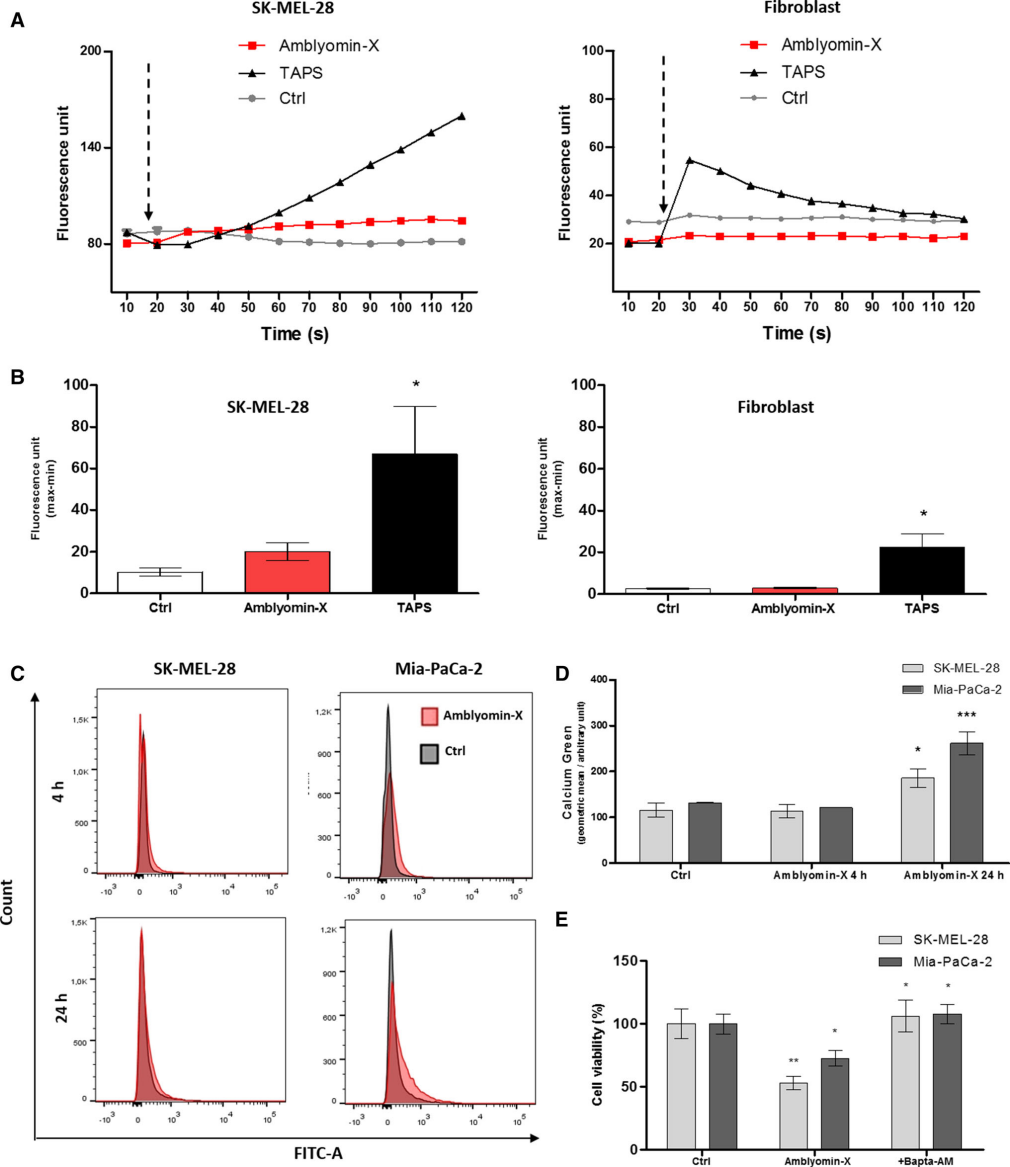
Amblyomin-X increases intracellular calcium levels in SK-MEL-28 cells. **a** [Ca^2+^]_*i*_ levels of unstimulated SK-MEL-28 and human fibroblast cells were measured for 20 s, followed by addition (marked by *arrows*) of Amblyomin-X (1 µM), TAPS (1 uM), or 50 uL of PBS 1X (Ctrl), and monitored for more than 100 s by microfluorimetry. **b** Graphic representation of quantitative measurements of fluorescence intensity (max–min) obtained from **a**. **c** Cells were treated with Amblyomin-X, stained with calcium green-1, and analyzed by flow cytometry. Representative histograms are expressed in fluorescence intensity. **d** *Bars graph* and geometric mean (fluorescence intensity) values ± SD obtained from C (three independent experiments). **e** Cells were pre-incubated for 30 min with BAPTA-AM (10 µM), followed by incubation with Amblyomin-X (1 µM) for 48 h at 37 °C

We further investigated whether treatment with Amblyomin-X would cause the mobilization of [Ca^2+^]_*i*_ in SK-MEL-28 cells using microfluorimetry. We observed a sustained but not a statistical increase in the [Ca^2+^]_*i*_, after the application of 0.5 µM of Amblyomin-X (Fig. 2a, b). In human fibroblast, no alterations were observed (Fig. 2a, b). In the positive control cells treated with TAPS (1 µM) a change in fluorescence signal was observed in both cell types. In Ctrl, cells were treated with PBS which showed no fluorescence alteration (Fig. 2a, b).

Next, we assessed the mobilization of [Ca^2+^]_*i*_ in SK-MEL-28 and Mia-PaCa-2 cells at 4 and 24 h after treatment of Amblyomin-X using fluorescence calcium Green-1 AM indicator in flow cytometry. The mobilization of [Ca^2+^]_*i*_ increased in both tumor cells after 24 h of Amblyomin-X treatment compared to control (Fig. 2c, d). The pre-treatment with BAPTA-AM protected the tumor cells from Amblyomin-X cytotoxicity (Fig. 2e).

### Amblyomin-X affect the mitochondria integrity

**Fig. 3:**
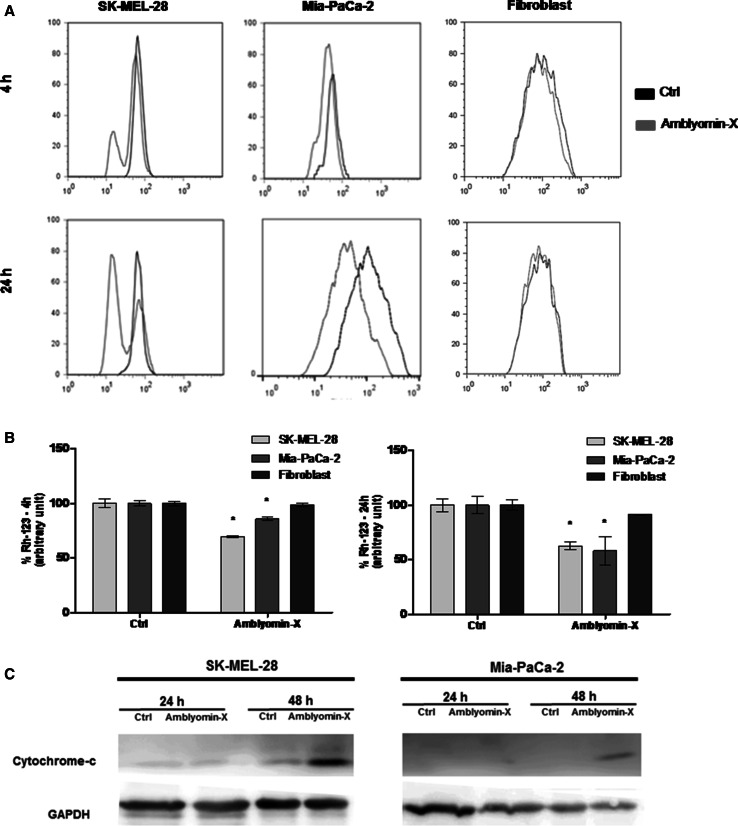
Mitochondrial dysfunction induced by Amblyomin-X in tumor cells. **a** Histogram representing the mitochondrial membrane potential. Cells were treated with Amblyomin-X (0.5 µM) for 4 h and 24 h. **b** *Bars graph* (fluorescence intensity) values ± SD obtained from **a** (three independent experiments). **c** After the indicated periods of treatments, cells were lysed. The cytoplasm and membrane were separated and 30 µg of cytoplasmic protein fractions was separated on SDS-PAGE. The Western blot of the samples was performed using anti-cytochrome-c and anti-GAPDH (endogenous control)

We investigated whether the Amblyomin-X causes mitochondrial dysfunction. In SK-MEL-28 and Mia-PaCa-2 cells treated with 0.5 μM of Amblyomin-X, the mitochondrial membrane changed slightly after 4 h. The mitochondrial membrane potential changed significantly in both cell lines after 24 h of its treatment with Amblyomin-X, but was more pronounced in SK-MEL-28 (Fig. 3a, b). Considering mitochondrial dysfunction induced by Amblyomin-X could result in the release of pro-apoptotic factors (such as cytochrome-c) into the cytoplasm, the cytoplasmic levels of the cytochrome-c were determined by Western blotting, which was increased after 48 h in the cell lines treated with 0.5 μM of Amblyomin-X (Fig. 3c).

### Caspase cascade activation in tumor cells by Amblyomin-X

**Fig. 4:**
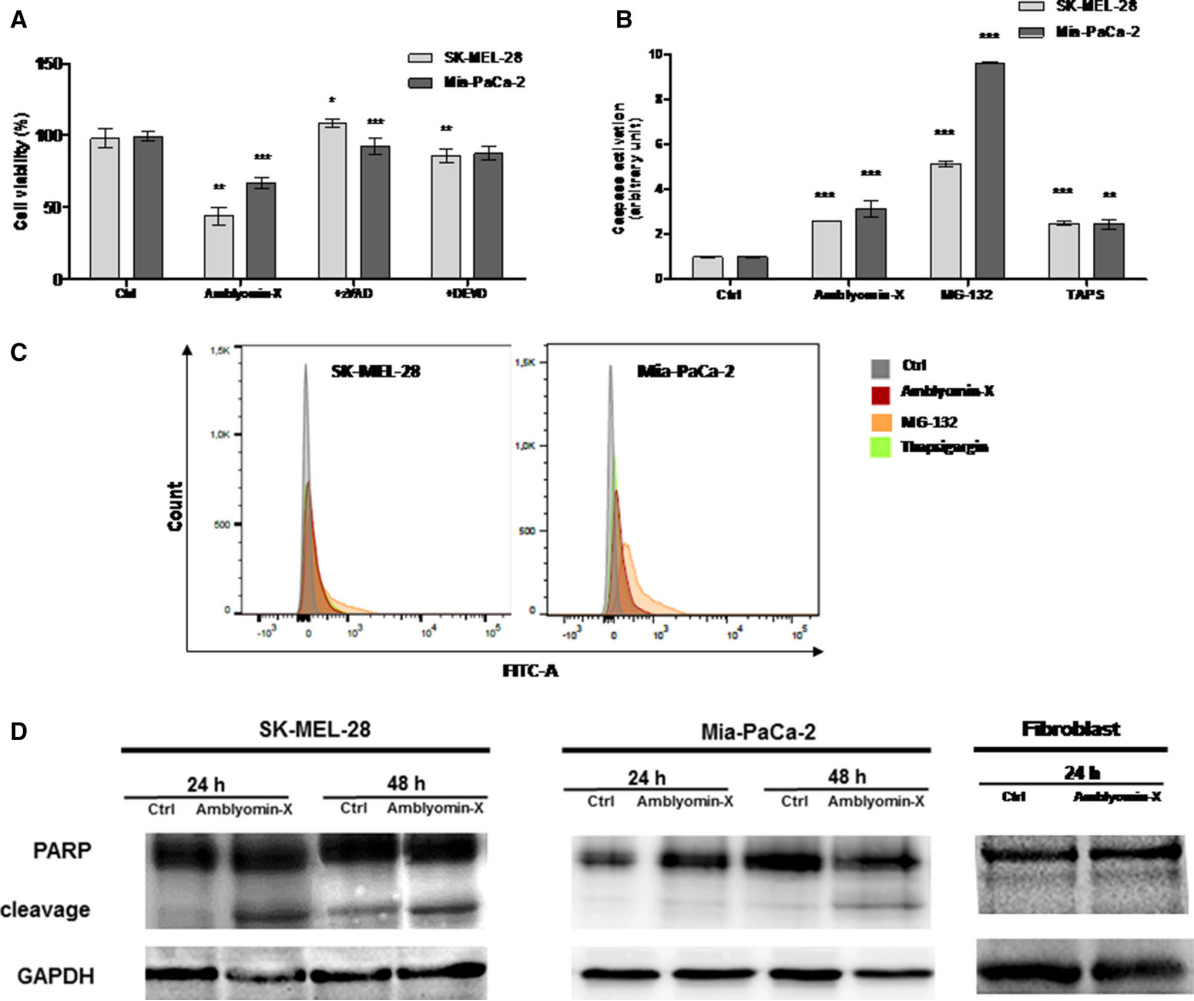
Caspase cascade activation after Amblyomin-X treatment in tumor cells. **a** Cells were pre-incubated for 2 h with ZVAD-FMK (50 µM) or DEVD-CHO (10 µM) followed by incubation with Amblyomin-X (1 µM) for 48 h at 37 °C. **b** Caspase activity was measured using CellEvent™ Caspase-3/7 Green Detection Reagent (Molecular Probes), according to manufacturer’s instructions. Cells treated with vehicle (PBS), or 0.5 µM of Amblyomin-X for 48 h, or with MG-132 (2.5 µM) and TAPS (1 µM) for 24 h each. Then, cells were stained with CellEvent™ Caspase-3/7 Green Detection Reagent and were analyzed by flow cytometer. **c** *Bars graph* (fluorescence intensity) values ± SD obtained from B (three independent experiments). **d** After the period of treatment, cells were lysed with RIPA buffer and 30 µg of protein extract was separated on SDS-PAGE. Western blot analysis were performed using anti-PARP and anti-GAPDH (endogenous control). *p ≤ 0.05, **p ≤ 0.01, and ***p ≤ 0.001 (Ctrl vs Amblyomin-X or Amblyomin-X vs inhibitors)

The release of cytochrome-c from mitochondria to cytoplasm causes the activation of caspase cascades via caspase-3 leading to apoptosis [32]. Thus, we pre-incubated tumor cells for 2 h with pan caspase inhibitor ZVAD-FMK. Subsequently, Amblyomin-X was added to the tumor cells and grown for further 48 h at 37 °C as discussed in materials and methods. Tumor cells overcome cytotoxicity of Amblyomin-X, bringing the viability to ~100 % in SK-MEL-28 and ~92 % in Mia-PaCa-2 cells (Fig. 4a). Likewise, when tumor cells were pre-incubated with caspase-3 inhibitor DEVD-CHO, cell viability was ~86 % in SK-MEL-28 and ~87 % in Mia-PaCa-2 cells. When those tumor cells were not pre-treated with caspases inhibitors, cell viability was ~45 % in SK-MEL-28 and ~60 % in Mia-PaCa-2 cells treated with 0.5 µM Amblyomin-X (Fig. 4a)

.

We also quantified caspase 3/7 activity measuring the fluorogenic response resulting from DEVD peptide cleavage. As shown in Fig. 4b, c, Amblyomin-X increased caspase 3/7 activity compared to negative controls. MG-132 and TAPS were used as positive control.

Next, we determined PARP cleavage using Anti-PARP antibody as discussed in materials and methods. PARP is a 116-kDa nuclear (ADP-ribose) polymerase involved in DNA repair predominantly in response to environmental stress [33]. This protein could be cleaved by caspase-3 and 7 [34, 35] facilitating disassembling of the cellular components and this serves as a marker for cells undergoing apoptosis [33]. We evaluated PARP cleavage in tumor cells treated with Amblyomin-X. A cleaved PARP band observed in SK-MEL-28 cell after both 24 and 48 h of Amblyomin-X treatment (Fig. 4d). In Mia-PaCa-2 cells, a faint PARP cleavage band was observed after 24 h, which becomes prominent after 48 h of Amblyomin-X treatment (Fig. 4d). In human fibroblast, cleaved PARP band was not detected (Fig. 4d).

## Discussion

Recently, it has been demonstrated that Amblyomin-X induces apoptosis in murine and human tumor cell lines [26, 27]. Herein, we demonstrated unprecedented results of Amblyomin-X cytotoxic effect on four tumor cells lines (Panc1, BxPC3, AsPC1, and SK-MEL-5). The amount of viable cells was different for both tumors cells treated with Amblyomin-X, which could be associated to the features of these cells. For example, Mia-PaCa-2 tumor cells derived from the pancreas adenocarcinoma of a 65-year-old man do not express measurable amounts of carcinoembryonic antigen [36, 37]. On the other hand, AsPC1 cells obtained from a 62-year-old woman produce abundant mucin as well as carcinoembryonic antigen [36, 38]. Human pancreatic cells sensitive to Amblyomin-X (Table 1) have mutation in the KRAS (v-Ki-ras2 Kirsten rat sarcoma viral oncogene homolog) and BxPC-3 is the wild-type exception [36]. Amblyomin-X sensitive human melanoma cells have mutant BRAF gene that increases kinase activity of BRAF, leading to constitutive activation of the RAS-RAF-MEK-ERK pathway and thus uncontrolled tumor proliferation [39, 40]. Obviously, the features of cells tested generate different levels of migration, invasion, tumorigenicity, and chemosensitivity. Despite that, all cells lines were sensitive to Amblyomin-X. SK-MEL-28 is sensitive to bortezomib [41], but Mia-PaCa-2, Panc1, and AsPC1 are resistant to this proteasome inhibitor [28]. In general, bortezomib-resistant cells overexpressed the β5 subunit (a target of bortezomib and have chymotrypsin-like activity) [42], which has a mutation in the binding site for bortezomib [42] or in downstream protein of the enzymatic complex [43]. In fact, Amblyomin-X inhibits the trypsin-like activity of the proteasome in tumor cells and indicates a similar interaction with β2 subunit, which is under investigation. Thereby, proteasome inhibition and/or other but yet unknown actions had made pancreatic cells sensitive to Amblyomin-X. These findings together with previous studies suggest that Amblyomin-X could function as a broad-spectrum antitumor drug. Of note, no alterations in cell viability of human fibroblast were observed, suggesting that Amblyomin-X is preferentially cytotoxic to tumor cells.

Here, we selected SK-MEL-28 and Mia-PaCa-2 in this study, since the role Amblyomin-X in protein clearance in these tumor cells have already been demonstrated [26, 29]. In fact, the data showed ER homeostasis disruption in tumor cells, since expressions of some genes of UPR and GRP78/GADD153 protein levels were elevated by Amblyomin-X treatment, as well as ATF-6α transport. An interesting point was the onset of ER stress responses (UPR gene expression at 2 h and GRP78 proteins levels at 4 h) in SK-MEL-28 cells prior to that induced via proteasome inhibition by Amblyomin-X. Thus, we cannot rule out the signaling independent proteasome inhibition. In fact, we need more investigation regarding ERAD process in tumor cells treated with Amblyomin-X. The changes in human fibroblast cells after Amblyomin-X treatment were different from those observed in tumor cells, i.e., the IRE1α and eIF2α levels increased but there was no modulation in GRP78/GADD153 levels.

In addition, perturbations of ER could compromise its function because it is one of the major intracellular Ca^2+^ storages. We observed no increase in [Ca^2+^]_*i*_ immediately after Amblyomin-X application in SK-MEL-28 cells (Fig. 2a, b), but not in human fibroblast cells. However, we noticed that the increase in [Ca^2+^]_*i*_ induced by Amblyomin-X was not transient, but proportional to the incubation time. In SK-MEL-28 cells and Mia-PaCa-2, this phenomenon was observed after 24 h of Amblyomin-X treatment (Fig. 2c, d). The blocking of Amblyomin-X cytotoxicity by BAPTA-AM indicates that calcium released from intracellular storage (ER, mitochondria) to cytoplasm plays an important role in mode of action of Amblyomin-X.

In addition, Amblyomin-X treatment promoted mitochondrial dysfunction only in tumor cells, indicating the involvement of the intrinsic pathway of apoptosis. Several antitumor drugs have been reported to work via activation of the intrinsic pathway of apoptosis [32]. The results supported our prediction because cytochrome-c released in tumor cells were treated with Amblyomin-X, however, only after 48 h of treatment. This suggests that no adequate response was achieved to generate drastic changes in mitochondrial potential in early periods of treatment (4 and 24 h), but that which intensifies over longer periods of treatment and later become sufficient to release this pro-apoptotic factor. The cytochrome-c released into cytosol and forms a complex with APAF-1 and pro-caspase-9, called the apoptosome, which promotes the cleavage of pro-caspase-9 and the release of active caspase-9 [44]. Once activated, caspase-9 activates caspase-3, which leads to apoptosis [45]. The activation of caspases could be involved in mechanism of action of Amblyomin-X, since its cytotoxicity was blocked by caspases inhibitors and Amblyomin-X treatment caused caspase-3/7 activation and PARP cleavage. However, in SK-MEL-28 cells, cleavage of PARP was detected prior to the release of cytochrome-c, suggesting the activation of caspase-3 pathway through other components, such as ER stress (caspase-4) and death (caspase-8) [32].

Even with all this evidence, it is not possible to establish a clear relationship between ER stress, elevations of [Ca^2+^]_*i*_, mitochondrial dysfunction, and caspase activation induced by Amblyomin-X. Notwithstanding, there is no way of ruling out the possibility of activation of these pathways separately by Amblyomin-X, and that their individual cellular response converge in apoptosis, or proceed until reaching the point of synergy among the pathways. However, more investigations are necessary to define the sequence of these events. In conclusion, these investigations might help in further understanding of the antitumor properties of Amblyomin-X, which has potential to become a promising candidate for the treatment of cancer.

## Conclusion

Many investments and efforts are made to improve cancer therapy; for example, in recent years, dozens of proteasome inhibitors have been synthesized and studied. Herein, we have demonstrated that Amblyomin-X, a proteasome inhibitor disturbs cell homeostasis and overlaps the tumor resistance mechanisms, culminating in cell death. Although cells upon Amblyomin-X treatment present differences in time and intensity of pro-apoptotic effects, the mechanism of action observed is similar in the tumor cells studied. Interestingly, all investigations on Amblyomin-X have shown its toxicity to tumor cells, but not to normal human fibroblasts cells, reinforcing its selectivity to tumor cells. Thus, Amblyomin-X could be a promising candidate for the treatment and study of cancer.

## Experimental section

### Amblyomin-X production

The homogeneous recombinant protein was prepared as reported elsewhere [25].

### Cell lines and culture conditions

Human’s melanomas (SK-MEL-28 and SK-MEL-5) and pancreatic adenocarcinomas (MIA-PaCa-2, Panc1, BxPC3, AsPC1) cells were obtained and cultured according to instructions of American type culture collection (ATCC, Manassas, VA). Human normal fibroblasts were isolated from biopsy dermal tissue of adult subjects. Cells were maintained in Dulbecco’s Modified Eagle Medium (DMEM) supplemented with 15 % fetal bovine serum and 1 % of antibiotic solution (0.1 mg/mL of streptomycin and 100U/mL of penicillin). All cell lines were routinely grown in a humidified 5 % CO_2_ incubator at 37 °C.

### Cell viability assay

Cell viability was measured by MTT tests as described elsewhere [24]. In order to investigate the caspase inhibitors or chelating intracellular calcium, SK-MEL-28 and Mia-PaCa-2 cells were seeded in 96-well plates (10^4^ cells/well) and pre-incubated with specific caspase-3 inhibitor DEVD-CHO (10 µM), pan caspase inhibitor ZVAD-FMK (50 µM), or with BAPTA-AM (10 µM) for 30 min. Finally, cell viability was measured by MTT assay.

### Quantitative real-time PCR analysis

Tumor cells (SK-MEL-28 and Mia-PaCa-2) and human fibroblast were harvested at various time points after treatment with Amblyomin-X. Subsequently, the total RNA was extracted from these cells lines using the RNeasy kit (Qiagen, Netherlands) and DNase treatment was carried out. Then, cDNA strands were constructed using a SuperScript^®^ III First-Strand Synthesis kit (Invitrogen™, Life Technologies Inc., USA), according to manufacturer’s instructions. Finally, the samples were applied in a SYBR^®^ green (Applied Biosystems, USA)-based reaction with specific and validated primers to evaluate mRNA levels of genes coding for ER stress proteins. The GAPDH (Glyceraldehyde-3-phosphate dehydrogenase) gene expression level was used as normalize control. The experiments were conducted by Step One Plus^®^ PCR real-time system (Applied Biosystems, USA) with the following cycle conditions: 10 min at 95 °C, followed by 40 cycles of denaturation of 15 s at 95 °C, 30 s at 60 °C, and finally 30 s at 72 °C. The relative mRNA levels of gene expression were calculated using the Pfaffl method [46].

### Indirect immunofluorescence

ER stress-induced transport ATF-6α was verify according previous reports [47]. Tumor cells were grown on coverslips in 15 × 15 mm 6-well plate and treated with Amblyomin-X (1 µM) for 4 and 24 h; or MG-132 (2.5 µM) for 24 h, a known proteasome inhibitor; or thapsigargin (1 µM) for 24 h, a selective inhibitor of endoplasmic reticulum Ca2+-ATPase, which was reported to induce ER stress. Cells were washed twice with 1X PBS and fixed with 4 % paraformaldehyde for 15 min at room temperature. The washing step was repeated and, then cells were incubated with a cell permeabilization solution (0.1 % saponin in PBS 1X) for 15 min at room temperature. Samples were washed and were incubated with blocking solution 1 % BSA for 30 min at room temperature. Following, the primary antibody was incubated overnight at 4 °C in (i) anti- human- ERGIC 53 mouse (Origen, Netherlands) 1:100; (ii) anti-human ATF-6α rabbit (Santa Cruz Biotechnology, Inc., USA) 1:50. A washing procedure was carried out and samples were incubated with secondary antibodies Alexa Fluor^®^ 555 goat anti-rabbit (Invitrogen™ Life Technologies Inc., USA), and Alexa Fluor ^®^ 488 rabbit anti-mouse (Invitrogen™ Life Technologies Inc., USA), both at 1:200 dilution for 1 h at room temperature in the dark. Cells were washed and one drop of solution anti-fade mounting VECTASHIELD^®^ (Vector Labs, USA) was applied on the slide with cover slip containing cells facing down and, then, sealed. The analysis was performed in LSMS Zeiss 510 confocal microscope (Zeiss, Germany).

### Western blot analysis

Total cellular proteins were extracted from tumor cells treated with Amblyomin-X or PBS 1X (Ctrl) using strong RIPA lysis buffer (150 mM NaCl, 1.0 % IGEPAL^®^ CA-630, 0.5 % sodium deoxycholate, 0.1 % SDS, and 50 mM Tris, pH 8.0.) and were quantified by BCA (bicinchoninic acid assay) using the Pierce^®^ Microplate BCA Protein Assay kit (Thermo Scientific, USA). Then, protein samples (30 µg) were subjected to standard SDS-PAGE and were transferred onto a polyvinylidene difluoride membrane (GE Healthcare, USA). For detection of protein of interest, primary polyclonal antibodies were applied on membrane. Primary antibodies used were anti-GRP78 (Santa Cruz Biotechnology, Inc., CA, USA), anti-GADD153 (Santa Cruz Biotechnology, Inc., USA), anti-PARP (Cell Signaling, MA, USA), and anti-GAPDH (Sigma-Aldrich, MO, USA). Bound antibodies were detected by chemoluminescence method *using* a homemade solution (1.5 M Tris pH 8.9; 20 mM *p*-coumaric acid, 125 mM luminol, and 30 % peroxide hydrogen). The images were acquired at each 10 s exposure in the LAS 4000 equipment (GE Healthcare, USA), employing the ImageQuant^®^ software.

### Preparation of protein extract for cytochrome-c measurement

SK-MEL-28 and Mia-PaCa-2 cells were treated with Amblyomin-X. Cells were harvested and were washed twice in PBS. For cytoplasmic cytochrome-c measurement, a modified extraction procedure was used compared to the one we employed for the analysis of GRP78, GADD153, and PARP. Briefly, to obtain a cytoplasmic proteins fraction, cells were re-suspended in relatively weak lysis buffer (HEPES–KOH 10 mM; pH 7.4, EDTA 0.1 mM; bis‐etilenoglicol (β‐aminoethyl ether); EGTA 1 mM; sucrose 250 mM; orthovanadate (Na3VO4) 1 mM, PMSF 1.0 mM, and dithiothreitol (DTT) 10 mM) and were incubated on ice for 15 min. Samples were then centrifuged at 10,000 g for 15 min at 4 °C to sediment unbroken cells, nuclei, and the heavy membrane fractions containing mitochondria or other cellular compartments. Supernatants containing mostly cytoplasmic proteins were collected and were subjected to immunoblot analysis using anti-cytochrome-c (Santa Cruz Biotechnology, Inc., USA).

### Measurements of changes in free intracellular calcium concentration ([Ca^2+^]_i_) by microfluorimetry

Changes in cytosolic calcium concentration ([Ca^2+^]_*i*_) were monitored by microfluorimetry, described elsewhere [48]. For this purpose, SK-MEL-28 and human fibroblast cells were transferred into 96 black-well plates (Costar, UK) at a density of 5 × 10^4^ cells per well in serum-free medium. Cells were then kept with FlexStation Calcium Kit containing 2.5 mM probenecid for 60 min at 37 °C. Before and after addition of Amblyomin-X or TAPS, fluorescence of samples was excited at 485 nm, and fluorescence emission was measured at 525 nm.

### Measurement of intracellular Ca^2+^ levels by flow cytometry

Tumor cells (SK-MEL-28 and Mia-PaCa-2) were grown with 0.5 µM Amblyomin-X or PBS for 4 and 24 h. Cells were washed with PBS, and incubated with 10 µM of calcium green-1-AM (Molecular Probes, OR). Calcium green-1-AM is labeled with calcium indicator molecule that changes fluorescence upon binding Ca^2+^ in the presence of 0.2 % of non-ionic surfactant pluronic acid F-127 (Sigma-Aldrich, MO, USA). Stained cells were examined on flow cytometer FACSCalibur (Becton, Dickinson and Company, CA, USA). For data analyses, FlowJo (FlowJo, LLC, OR, USA) software was employed.

### Mitochondrial potential measured

The rhodamine 123 (^123^Rh) is a lipophilic cationic fluorochrome that is readily incorporated into mitochondria in manner-dependent mitochondrial membrane potential [49, 50]. It is a cationic fluorochrome, which is attracted by a high negative electrical potential present in the mitochondrial membrane. Changes in mitochondrial membrane integrity cause diffusion of ^123^Rh from mitochondria to cytosol and it can be detected by a decrease in fluorescence [49, 50]. Briefly, after 4 or 24 h of Amblyomin-X treatment (0.5 µM), the tumor cells (SK-MEL-28 and Mia-PaCa-2) and human fibroblast cells (10^5^ cells/plate) were suspended in 300 μL FACs buffer and incubated with ^123^Rh probe (200 ng/mL) in a humidified 5 % CO2 incubator for 30 min at 37 °C. Finally, cells were washed in cold PBS, and mitochondrial potential was measured using a flow cytometer FACSCalibur (Becton e Dickson, CA, USA). For data analyses, FlowJo (FlowJo, LLC, OR, USA) software was employed.

### Caspase activation

To verify caspase activity, CellEvent™ Caspase-3/7 Green Detection Reagent (Molecular Probes Inc. USA) was used according to manufacturer’s instructions. Briefly, tumor cells were treated with Amblyomin-X (1 µM) for 4 and 24 h; or with MG-132 (2.5 µM) for 24 h; or with TAPS (1 µM) for 24 h. Then, tumor cells were stained with CellEvent™ Caspase-3/7 Green Detection Reagent for 30 min at 37 °C. The fluorogenic response resulting from DEVD peptide cleavage was measured by flow cytometer FACSCalibur (Becton e Dickson, CA, USA). For data analyses, FlowJo (FlowJo, LLC, OR, USA) software was employed.

### Statistical analysis

Comparisons were carried out using Two-way ANOVA analysis followed by Tukey’s Post Hoc test or t-Test, employing the GraphPad Prism 5.0 software (GraphPad Software Inc., San Diego, CA). The criteria for statistical significance were set up as *p ≤ 0.05, **p ≤ 0.01, and ***p ≤ 0.001.

## Abbreviations

ER: Endoplasmic reticulum
[Ca^2+^]_*i*_: Intracellular calcium concentration
ERAD: ER-associated degradation
UPR: Unfolded protein response
GRP78: 78 kDa glucose-regulated protein; kinase-like ER kinase (PERK), the inositol requiring kinase 1 alpha (IRE1α), and the transcription factor-activating transcription factor 6 (ATF6)
eIF2α: Alpha subunit of translation initiation factor
GADD153: Nuclear transcription factors of growth arrest and DNA damage
TRAF2: Tumor necrosis factor TNF receptor-associated factor (TRAF) 2
JNK: c-Jun-*N*-terminal kinase
^123^Rh: Rhodamine 123
TAPS: Thapsigargin
PARP: A 116 kDa nuclear poly (ADP-ribose) polymerase
TFPI: Tissue factor pathway inhibitor
TF: Tissue factor
KRAS: v-Ki-ras2 Kirsten rat sarcoma viral oncogene homolog
PERK: Protein kinase-like ER kinase
IRE1α: Inositol requiring kinase 1 alpha
ATF6: Transcription factor-activating transcription factor 6
ATF4: Actor-activating transcription factor 4
